# Electrode Materials in Microfluidic Systems for the Processing and Separation of DNA: A Mini Review

**DOI:** 10.3390/mi8030076

**Published:** 2017-03-03

**Authors:** Christopher Birch, James P. Landers

**Affiliations:** 1 Department of Chemistry, University of Virginia, Charlottesville, VA 22904, USA; cb2xj@virginia.edu; 2 Department of Mechanical and Aerospace Engineering, University of Virginia, Charlottesville, VA 22904, USA; 3 Department of Pathology, University of Virginia, Charlottesville, VA 22904, USA

**Keywords:** electrode, microfluidic, DNA, electrophoresis, separation

## Abstract

Since the advent of genetic analysis, electrode materials have played an irreplaceable role due to the easily-exploitable negatively-charged backbone of the DNA structure. Initially, the employment of electrophoretic movement lay only in the separation of DNA fragments of differing length; however, the widening utility of electrokinetic phenomena at the microscale in areas such as fluid transportation and multistep integration led researchers to capitalize further when translating processes to microfluidic or “lab-on-chip” devices. Over the following three decades, the field witnessed a plethora of ways in which the necessary voltages could be transmitted to the sample and reagents with many successes; however, additional demands were then placed on those hoping to bring their microdevices to the market place. A greater emphasis on the cost of all constituent parts along with the increased importance that fluidics be contained hermetically at all times meant groups would become more imaginative when incorporating electrode materials. This review will aim to exactly describe the evolution of how those materials have been employed in DNA-based microfluidic devices. It will focus on how developers began to explore other emerging uses and also discuss how their tactics reflected the progressive demands of their chosen industry.

## 1. Introduction

Microfluidic technologies have progressed significantly over the last three decades, with those in the field reporting remarkable innovations in engineering, fluid dynamics, and in both the biological and chemical sciences [1]. As the field first garnered interest, pioneers placed upon themselves the challenge of delivering an analytical platform which could miniaturize established large-scale processes, thus reducing reagent usage, lowering costs, and expediting the analysis in its entirety. In addition, the microfluidic platform held the potential for integrating multiple steps by eliminating the invasive nature of transferring a sample between stages manually. Protocols such as these are generally labor-intensive and must be accommodated by several work stations within a laboratory. Furthermore, both the sample and the operator are put at greater risk of compromise or contamination, necessitating a contained laboratory environment, which precludes in situ analysis [2].

In 2016, it is now realistic to say that the devices or “chips” being produced are performing close to the level promised when the field was still in its infancy. However, while researchers are demonstrating greater knowledge, expertise, and control over operations within the microfluidic domain, there are still many hurdles awaiting those hoping to bring an integrated device from the laboratory to a commercial setting [3]. One leading field of interest that has attracted many researchers is that of nucleic acid analysis, both in the clinical and forensic realms [4,5,6]. From a clinical perspective, this can concern the isolation of mutations within the human genome to diagnose various pathologies (e.g., cancer) or can aid in the detection of infectious pathogens like bacteria and viruses [7]. Forensic human identification, however, is geared towards the characterization of human samples by targeting genetic markers that distinguish one individual from another [8]. Both are contingent on the amplification of an intended target with the polymerase chain reaction (PCR), where a combination of target probes and constructive enzymes are used to replicate and multiply the number of copies of a target in an exponential manner. This, in turn, is normally preceded by a sample preparation or clean-up step, which renders the target DNA free from inhibitors and amenable to amplification [7,9]. Detection of amplified genetic analytes can be carried out in one of several ways, including by electrochemical, chemiluminescent, or fluorescent detection. For clinical applications, this can often be a qualitative detection of a single or low number of targets. However, concerning the ‘genetic fingerprinting’ of a human subject, the process must isolate up to 20+ targets and then display them in a lucid fashion. This demands that the detection step be expanded to include efficient separation of individual genetic markers.

The DNA molecule possesses structural advantages as an analytical target, offering an easily exploitable negatively-charged backbone throughout its double helical structure [7,9]. This property has enabled the manipulation of DNA by exploiting its inherent electrophoretic mobility through a sieving matrix provided by a gel or polymer network [10]. Electrokinetic mechanisms, such as this, predate microfluidic technologies by some 50 years and have underpinned the broader field of separation science [11,12]. Conventionally, migrating species are directed through the sieving matrix, causing individual migration times of particles to be protracted to varying degrees based on unique structural and chemical interactions with the surrounding material. It is this property that allows the species to be separated and, with the appropriate labeling chemistry, observed. It was first manifest in the form of gel electrophoresis, most commonly adopting a slab of agarose gel as the separation matrix, followed by subsequent visualization of migrating species with a fluorescent dye, such as ethidium bromide [13]. Electrophoretic separation science then underwent significant augmentation when the separation format was transferred from slab gel to a gel-filled capillary. Here, a microscalar capillary is filled with a separation polymer, reducing processing times and improving resolution of the separated components [14,15]. Due to the major commercial interest surrounding capillary electrophoresis (CE), the comparable dimensions employed in microfluidic applications were found to be amenable to similar molecular transport mechanisms. Some regard this as one of the key transitions that ignited widespread interest in the then conceptual “micro total analytical system” (µTAS), a concept which evolved into the complex multistage microfluidic device that is sought after today [1].

As µTAS grew more complex, established fluidic concepts derived from electrokinetic phenomena were unveiled as applicable and implemented by researchers in unique and imaginative ways. Electroosmotic flow (EOF) (the bulk flow of fluid driven by the electric double layer at the fluid/channel wall interface) was adopted by various groups for transporting sample and reagents [16,17,18]. Inspired by the efficacy of EOF, several groups would then implement its mechanistic offspring, electroosmotic pumping (EOP). This was generally aided by high surface area structures such as porous materials, of which synthesis constituted part of device preparation. Finally, dielectrophoresis uses a similar setup to induce a non-uniform electric field within a channel, allowing polarizable particles, such as cells and marker particles to be transported [19,20]. These concepts are elegantly described in the following articles [2,21,22].

In order for genetic analysis to be driven by electrokinetic transportation mechanisms, the inner fluidics must interact directly or indirectly with a power supply necessitating some form of electrode material. These fluidics may comprise a buffered or electrolyte solution or a gel, such as agarose or polyacrylamide. The integration of electrode materials in this way, as with the introduction of any other additional components, means the microfluidic device grows more complex and more costly to manufacture [1]. Considering this, electrode manufacture and incorporation is most efficient if both are performed at the same time, with additional advantages if it is carried out in a manner derived from the primary fabrication method. This enables greater control of size, shape, and position, which minimizes any variability introduced by the user and maximizes reproducibility at time of use [23]. Experimental fluctuations felt by this margin of error are more pronounced when integrating electrodes for electrochemistry-based applications; however, these effects must not be ignored when employing more simplistic electrokinetic mechanisms, as performance can often be influenced by the position of the electrode relative to the sample and chamber features.

A holistic manufacturing strategy, as described, serves to limit any unnecessary interactions between the user and/or instrumentation and the inner fluidics, which is essential in ensuring sample and reagents remain free from contamination. This promise to eliminate contamination is invariably a target, however, should the end-goal be device commercialization, its importance is elevated further [24]. Considering the employment of PCR in the realm of forensic and clinical applications: here, each result is entirely dependent on a clean and reliable sample. Otherwise, the validity of data can be compromised. For instance, the presence or potential presence of contaminating human genomic DNA in a forensic scenario can often sully otherwise reliable data and, regarding clinical diagnostics, cross contamination can lead to false positives, resulting in misdiagnoses [7,8]. These stringent rules regarding sample integrity, therefore, forbid the invasion of the device inner-architecture with instrument components, such that any interference by an external electrode or power supply would threaten to vitiate analysis performed on the device. For this reason, it is now preferred that microfluidic devices dependent on electrical forces are integrated with electrodes to channel any desired current between an external power source and the inner architecture. This bridging material equips the user with the same level of control while the device itself remains hermetically sealed, shielding the sample and reagents from potential contaminants [25].

Those developing DNA (and other) separation microfluidic devices are primarily concerned with features that determine the processing timescales, sensitivity, and resolution quality of separated fragments. However, the requirement for suitable and cost-effective electrode materials is an additional factor, which must also be considered throughout development, rather than as a final add-on. The aim of this review is to unearth and expound any cases where electrode material was incorporated to act purely as a bridging material rather than to perform complex functionalities such as electrochemical detection or resistive heating. While its role may seem trivial, its importance is becoming greater as more devices are manufactured for field work. It is also important to note that those integrating electrodes with other functionalities are not always burdened with the same onus to provide full hermetic containment. That being said, the reader is directed to the following articles for a greater coverage of those areas [26,27,28]. The review, first and foremost, will draw from DNA separation-based applications; however, the work of those developing alternative separation microdevices, but share the same goal, will also be included. The document will be exclusively concerned with the development and incorporation of integrated electrodes from a fabrication perspective. In providing a comprehensive list of reported work, the authors hope to equip the reader with the knowledge to select an appropriate electrode type for their device or application.

## 2. Integrated Electrodes

The term electrode was coined by William Whewell in the 1850s, in collaboration with Michael Faraday [29]. While the word now has several nuances within the field of electronics and electrochemistry, its broader usage applies throughout this article, denoting a conductive material that allows current to flow to and from a non-metallic medium. Predictably, certain materials are more suited to achieving this than others, and selection often involves a trade-off of relevant pros and cons. Characteristics considered when selecting an electrode material are conductivity, reactivity or inertness, cost, and malleability. Gold and platinum are considered leading materials due to a combination of conductivity and inertness, with users willing to pay approximately $900–1200 per ounce. Silver is more conductive at a significantly lower cost (<$50 per ounce). However, it is more reactive and can become tarnished over time. These prices reflect the Bloomberg index as of October 2016. The work described in this review will predominantly comprise Au and Pt. However, several other elements, e.g., Ag, Cu, stainless steel and carbon, also appear. Additional information about these preferred metals is available in Table 1.

The broader area of electrochemistry may demand that users evaluate additional material characteristics such as surface area, electrochemical activity, and its amenability to repeated use. Considering this, separation devices may also be susceptible to undesirable electrochemical effects at the electrode surface, notably the formation of bubbles. While an in-depth exploration of these effects is beyond the scope of this review, the issues of bubble formation in electrophoresis microfluidic devices by electrolysis have been both reported and tackled, so developers must remain vigilant throughout testing [30,31,32].

**Figure 1:**
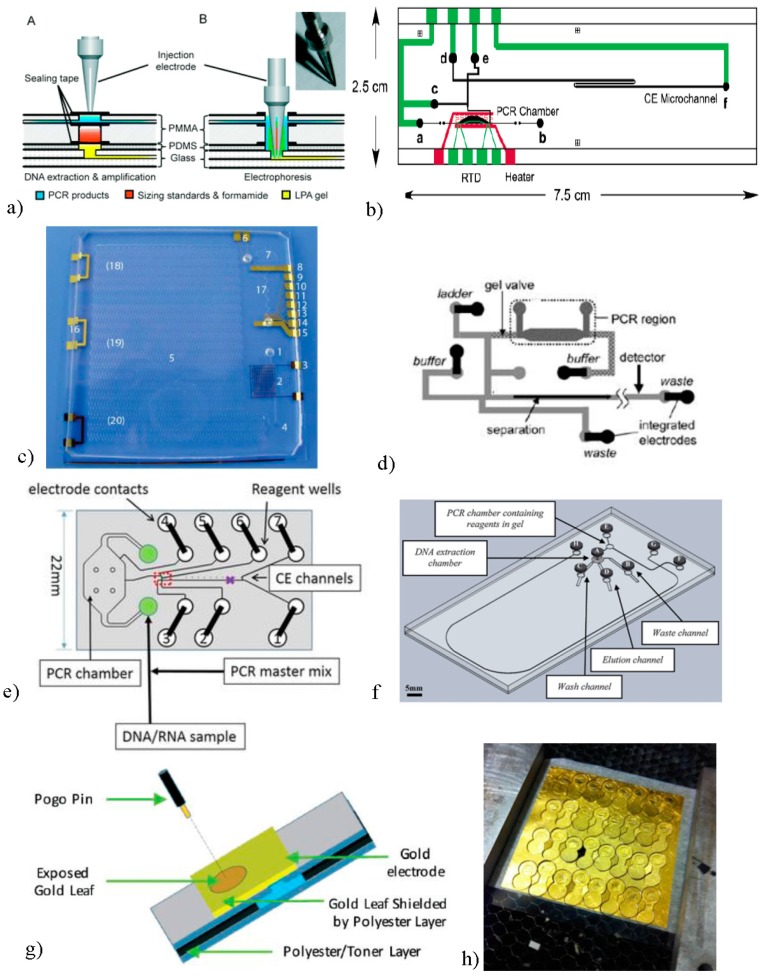
Integrated electrode examples. (**a**) Reproduced with permission from [36]; published by Royal Society of Chemistry, 2016. (**b**) Reprinted with permission from [49]; published by American Chemical Society, 2004. (**c**) Reproduced with permission from [52]; published by Royal Society of Chemistry, 2012. (**d**) Reproduced with permission from [54]; published by American Chemical Society, 2003. (**e**) Reproduced with permission from [55]; published by Wiley, 2016. (**f**) Reproduced with permission from [56]; published by Royal Society of Chemistry, 2011. (**g**,**h**) Reproduced with permission from [25]; published by Royal Society of Chemistry, 2016.

Established genetic CE instruments, e.g., the Applied Biosystems^®^ (ABI) 310 Prism Genetic Analyzer (Platinum) [33], Agilent 2100 Bioanalyzer (Platinum) [34] and Beckman Coulter^®^ Genome Lab™ Genetic Analysis System (stainless steel) [35] introduce electrodes mechanically and vertically to corresponding wells. The accompanying manuals contain extensive information about cleaning the electrode, dealing with damaged or bent electrodes and describe other electrode-based maintenance protocols. ABI advises that, due to crystallization at the electrode surface, the electrode should be cleaned every 48 h. Unfortunately, as modern microfluidic applications are often explored to aid fieldwork and in situ analysis, they do not facilitate frequent maintenance. Furthermore, instruments such as these are ample in that they can accommodate greater complexity to support instrument mechanics that can partition fluidic regions, thus limiting contamination. This is, however, more difficult to achieve with a microfluidic device, where a separation domain would represent a final analytical step downstream of sample preparation steps in an integrated system. A number of approaches have been described for introducing voltage to microfluidic systems in this way, with some success. Zhuang et al. describe a multilayer seal in which a Pt-coated steel electrode with a cross groove pierces the device, layer by layer, sequentially mixing amplified target and separation reagents as each layer is breached. The injection and separation steps are then achieved by actuating the electrode (Figure 1a). This device was used to identify single nucleotide polymorphisms that affect the metabolism of the anticoagulant, warfarin [36]. Similarly, Hopwood et al. introduced a polycarbonate plate containing gold-coated pins, which would slot into a reusable CE device. Here, contamination is minimized by sequential flushing of device components, akin to the regeneration of a capillary for sequential DNA separations in CE [37]. While these two approaches exemplify clearly how a sample can be sufficiently contained, the dependence on reusable components which are supported by cleaning steps in between runs derives from those protocols associated with commercial CE instruments. This approach is nonviable as the paradigm moves to a preference for fully-functional microfluidic devices that are single-use disposable (or recyclable). Consequently, techniques for integrating electrodes that meet this criteria have also been explored.

The first significant shift of DNA analysis processes to microfluidics can be traced back to 1992. During this period, Huang et al. took steps in tackling the limitations of CE at the time. The approach taken was to develop a platform for parallelized CE using bundles of capillaries, each accommodating separation of an individual sample [38]. This progress would inspire further exploration into CE and how its efficiency may be enhanced—a collective pursuit which was intensified by the recent revelation that photolithography could be employed as a means to fabricate glass microdevices. The combination of producing intricate architecture by way of wet etching, and subsequent high-temperature bonding, afforded researchers the ability to customize architecture to accommodate their applications, subsequently leading to a series of seminal papers on microchip separation that laid firm foundations to the following two decades of progress. The advances made would successfully outline effective channel architecture, optical properties, and optimal voltages [2].

The first report of electrophoresis on a microchip was made in 1992, when Harrison et al. demonstrated successful separation of fluorescein and calcein [39]. This principle was given greater utility a year later when the same group achieved separation of seven amino acids on similarly designed glass devices [40]. In 1994, Wooley et al. were able to translate this to genetic applications, using glass devices to separate DNA fragments between 70 and 1000 bp [41], expanding further by integrating PCR and subsequent separation on a single device [42]. Throughout this period, application of voltage was achieved by inserting Pt wire into designated wells attached to the device, an approach which would become a frequent recourse for those developing microelectrophoresis devices, including those for DNA. Of those devices described, only the former work of Harrison et al. [36] would propose a way in which platinum materials could be permanently integrated to the devices themselves. This entailed the integration of 20 µm Pt leads by bonding them between the two glass plates. The group does elaborate on difficulties with leakage around the electrodes due to hindered bonding. However, this was overcome and successful separation was reported. Another notable attempt to permanently integrate Pt wire into a device was that of McCormick et al. when adopting an acrylic copolymer device. Here, 76 µm diameter platinum wire was positioned between buffer regions and the edge of the device, enabling insulated electrical connection from that position [43]. Similarly, Sanders et al. [44] demonstrated an embedding of Pt wire into polydimethylsiloxane (PDMS), whereby the wire provides an electrically-insulated pathway from the separation domain to an accessible well for the application of voltage. Unfortunately, the high cost of platinum wire does undermine its use as an integrated disposable component and, consequently, demands its reuse as an external electrode, where it is repeatedly brought into contact with the internal fluidics of multiple devices, whether regenerated for reuse (akin to CE) or new devices. This would also likely introduce some variability when dipping the wire into the wells manually, as position within the well can be important and may be difficult to replicate in sequential separations [23]. Despite its limitations, however, Pt wire has proven to be extremely useful and allowed separation and detection technologies to evolve at an increasing pace unfettered. It is also presumed that those presenting more recent alternatives, discussed throughout this review, would continue to employ Pt wire as a temporary means throughout device development.

Exploration into alternatives for integrating electrode materials into electrophoresis systems began in the mid-90s. The attractive combination of CE and electrochemical detection encouraged many to fuse the two processes into one domain. However, much expertise was required when aligning electrode materials with the end of a capillary structure. Zhong et al. [45] attempted to solve this problem by wrapping gold wiring around the end of the capillary with some success. However, the fabrication protocol for this still demanded a great level of precision and expertise. Acknowledging this, Voegel et al. [46] offered a more practical alternative by sputtering Pt or Au material on to the ends of capillaries for both electrophoresis and electrochemical detection. The Au or Pt region could then be connected by Cu wire to a power supply. Previously, this metallization of glass surfaces had been utilized in the production of electronic devices, whereby an Au or Pt electrode layer is bonded to the glass, assisted in bonding using a transition metal film, such as Ti or Cr. Much like the photolithography used in etching glass devices, the electrode material can be deposited on a surface that is coated, in part, by a sacrificial layer. When this layer is removed, the electrode patterns remain. This deposition of metal onto the substrate can be carried out in one of two ways. Sputtering, as mentioned, involves the bombardment of a metal target material with high energy particles, causing deflection of atoms on to a nearby substrate. An alternative to this is thermal evaporation in which the target material is subjected to high temperatures, causing it to evaporate and engulf an adjacent substrate. In 1998, Burns reported microelectrophoresis of PCR product with fluorescence detection using electrodes integrated with this form of thermal evaporation [47]. This was achieved by depositing Pt on to a glass substrate prepared with a sacrificial mask defining the electrode region. By contrast, Woolley et al. employed sputtering when developing a device for separation and electrochemical detection of DNA fragments. However, the sputtered Pt was utilized only for the latter and the use of Pt wire was retained for the application of electrophoresis currents [48]. Interestingly, it was not until 2002 that Baldwin et al. brought these two concepts together to sputter Pt electrodes for both electrochemical detection and electrophoretic separation [23]. In that work, the sputtered Pt led to conductive bonding pads protruding from the edge of the device, which could be clipped to a power supply to apply the separation voltages with relative ease. In 2004, Lagally et al. sputtered Pt onto glass devices to carry out PCR and electrophoresis of genetic targets for detection of pathogens [49]. Adopting a different strategy, the Pt was sputtered first before a photoresist could be laid on top. This then allowed for removal of all non-electrode regions and subsequent etching away of the exposed Pt with aqua regia. Upon final removal of the remaining photoresist, the electrodes were left in their desired configuration. These electrodes were also paired with spring-loaded electrical contacts or “pogo pins” which connect on the edge of the device with a clamp (Figure 1b). Spring-loaded electrical contacts are popular with microfluidic applications as they can be lowered onto the device, while allowing for some freedom of error with distance and pressure. More recently, Floris et al. presented an elegant point-of-care device that combined electrophoretic separation and conductivity measurement of lithium in blood. Here, all electrodes were fabricated by deposition of Pt on an adhesion layer within a glass device, which could remain entirely contained throughout processing. This device, the Medimate Multireader^®^, is now commercialized [50]. While both sputtering and thermal evaporation effectuate adequate deposition, the former technique proved more suited as the directional nature of an evaporating material is not conducive to widespread coverage, thus requiring greater control during execution. For this reason, sputtering has since been the more prominent technique of the two for fabrication of electrodes.

As lab-on-chip technologies evolved, various polymeric substrates would gradually become more popular following the initial surge of interest in glass. Pourable elastomers, such a PDMS could be employed by casting in a silicon mold to fabricate device architecture, proving very useful as a prototyping material. It was well suited to biological applications, such as cell culturing, due to its high gas permeability, as well as being exploited to form mechanical valving structures [51]. As discussed earlier, Sanders et al. were able to embed Pt wire into PDMS/glass hybrid DNA analysis device. Jha et al. exploited a similar construct to implement electrodes on a glass substrate using a combination of photolithography and evaporation, before adding a layer of PDMS, which contained all necessary channel architecture [52]. Remarkably, the electrodes supported all steps, from cell lysis through PCR heating and CE, enabling separation of both human and bacterial targets (Figure 1c).

Cyclic olefin coploymer (COC) and various other thermoplastics (e.g., polymethyl methacrylate (PMMA)) have become increasingly popular for the manufacture of microfluidic devices. Unlike PDMS, thermoplastics can be utilized for injection molding or embossing the microfluidic architecture. This is more practical for mass-manufacture of devices and has been adopted by many groups when in the final stages of product development [51]. When fabricating fully-integrated DNA analysis devices for human profiling, Le Roux et al. [53] sputtered Au to lay electrodes for the separation step. Au was sputtered between two injection-molded COC layers, comprising an all-COC device. Voltage was applied to sputtered Au electrodes using spring-loaded pogo pins.

While electrode manufacture using metal sputtering and lithography has yielded effective DNA separation devices, the technique has become less prominent in recent years. This is partly due to the decreasing interest in glass-based microfluidics but can also be attributed to the cost of Pt and Au, when incorporated in this way. Sputtering (or evaporation) has desirable characteristics; however, the target material required to compose the electrode can cost in excess of $10,000 (USD), amounting to $10+ per device for electrodes alone. In addition, the cost per device typically correlates with its complexity, as the necessary stenciling technique contributes to higher levels of material waste. Unfortunately, as implied by lab-on-a-chip, replicating the processes/chemistry that we execute at the bench inevitably drives up the complexity of these integrated analytical microdevices. Combined, these factors can encumber manufacture with costs comparable to Pt wire. Considering this, while technologically impressive, the work of Le Roux is an example of how the cost benefits reaped by moving to more inexpensive polymers can be offset somewhat by the expense of additional components. This device was, however, an important milestone in providing a sample-to-answer electrode-based DNA analysis device that was fully contained and disposable.

An inexpensive alternative to lithography and sputtering techniques is that of screen printing. This approach, which dates back millennia, employs a woven mesh to transfer liquid materials to a substrate. By layering the mesh with a stencil, a desired pattern can be imprinted with precision and reproducibility. While the popularity of this technique lies firmly in designing of fabrics and, to some extent, fine art (Andy Warhol is a pioneer), it allows for the effective transfer of conductive ink, which can serve as an electrode material [57]. The electrochemistry community has reported carbon, Ag, Au and Pt electrodes screen printed [58]. In 2003, using a poly(cyclic olefin) device, Koh et al. reported a screen-printed Ag/graphite electrode for the electrophoretic separation of DNA fragments [54], as well as using the electrodes to support a resistive heater for PCR. To achieve this, the Ag/graphite ink was printed onto an additional poly(cyclic olefin) film and cured at 95 °C before being aligned with a primary device composed of the same material. In doing so, they were able to detect bacterial targets in the form of *E. coli* and *Salmonella* (Figure 1d). Similarly, Liu et al. [55], integrated screen-printed carbon electrodes by printing on a polycarbonate film for detection of *E. coli*. Here, the device was able to separate fragments electrophoretically, aided further by electrophoretic transportation of the amplicons from PCR chamber to the separation domain. The connecting well lay separate from sample or reagent wells, and the two are electrically-connected (Figure 1e). Screen-printed electrodes have not been utilized substantially outside electrochemical biosensing. One of the reasons is the incompatibility of the conductive inks with organic solvents, which can cause potential issues with durability on rigid surfaces. This is, perhaps, why screen-printing has cemented greater popularity in the field of paper microfluidics [59,60,61].

Invoking, again, the work by Lui et al. [55], this is an early and important example of how some had begun to employ electroosmotic flow in DNA devices. In 1993, the Manz group had thoroughly discussed electroosmotic flow as a means for movement of fluid around microfluidic devices [62], and posited that it could serve neatly as a sample or reagent transportation mechanism. Using sputtered Au electrodes on a glass/PDMS hybrid, McKnight et al. [17] demonstrated this form for a fluid transportation device. The work emphasized the advantages of using PDMS to allow any forming bubbles to permeate from the device. Oakley et al. [18] were able to employ electroosmotic pumping in a glass device to transport sample and reagents for DNA extraction using a silica monolithic structure. This was first achieved by exploiting Pt wire; however, the same group carried out similar operations using injection-molded carbon fiber-coated polystyrene electrodes. These electrodes were placed in reservoirs in the microfluidic device before voltage was applied using pogo pins attached to electrodes fitted into a custom-built instrument. Shaw et al. used this method in an integrated DNA extraction and amplification device for human profiling, again employing EOP for DNA extraction and transporting the sample between the two steps [56] (Figure 1f). Over the last half decade, EOF and EOP have not been reported extensively, possibly due to difficulties incorporating with other microfluidic features, such as valving structures. However, due to the ease of implementation, we may see these mechanisms return to DNA applications.

Much like the polystyrene electrodes employed by Oakley et al., conductive polymers are becoming more prominent in microfluidic applications. Bengtsson et al. [63]. recently reported use of pi-conjugated polymer electrodes in traditional gel electrophoresis methodology to separate multiple proteins. The key benefit discussed is a reduction in cost, which enables disposal and eliminates the risk of cross-contamination by reusing electrodes. Using a technique termed “flash welding”, Henderson et al. were able to fabricate an all-polymeric device with integrated polyaniline electrodes for microdevice electrophoresis [64]. Here, the desired electrode pattern can be transferred to a conductive polyaniline film by exposing the film to the correct radiation to disable the material’s conductivity. In doing so, the discrete zones, which are shielded, remain conductive and can be patterned, so they can align with separation architecture of a device and transmit current for electrophoresis [65]. This group demonstrated this with the separation of three sugars. Both of these works emphasize how the polymer electrodes performed comparably with Pt wire; however, the latter do report some loss of conductivity over time at high voltages, which resulted in limitations being placed on experimental parameters. Despite this, the ability to mold conductive polymers in this way implies that they could soon play a part in DNA separation and, considering flash welding in particular, offers a facile approach to manufacturing electrodes as part of the whole process. Moreover, there have been several reports of screen-printed conductive polymer electrodes within the electrochemistry community, perhaps offering another solution to integrating into electrophoresis microdevices.

The leading materials of choice for device fabrication featured thus far, while dominant, have been supported by several other cost-effective and imaginative techniques, ranging from paper, wax, felt pens and various easily-accessible polymers. Many of these materials have provided a useful platform for qualitative testing, such as colorimetric assays. However, they lack the properties to support robust fluid dynamics and integration. By contrast, do Lago et al. contributed a method for fabricating devices that consists of printing toner on to polyester transparency sheets to define microfluidic architecture [66]. Once aligned with other polyester sheets, multiple layers can be bound by lamination. This technique would later evolve into the print, cut, and laminate (PCL) technique described by Thompson et al. [67]. Building on previous work [68,69] and inspired by those toner-based techniques, Daniel et al. developed electrode materials using conventional recordable CDs. These CDs consisted of a polycarbonate substrate layer with a 50–100 nm sputtered Au layer, protected by a polymer layer. Following easy removal of the protective layer with nitric acid, like with polyester transparency sheets, toner is deposited on to the disc, followed by removal of the exposed gold via etching using aqua regia. After the toner is removed, the gold electrodes remain [70]. In 2016, Foguel et al. adapted and characterized this method further, by introducing a polytetrafluoroethylene (PTFE) layer to supplant the toner, facilitating the immobilization of various biological molecules [71]. These groups have used this approach for electrochemistry applications, but the method, thus far, has not been used to integrate electrodes for electrophoretic separations. For similar applications, nanoporous gold leaf (NPGL) has been synthesized and implemented into microfluidic devices to augment electrochemical performance by increasing surface area over already existing electrodes. This technology was employed by Ciesielski et al. to immobilize photoactive proteins for electrochemical detection [72]. This involved the de-alloying of a gold/silver leaf hybrid in nitric, and the subsequent mounting onto a secondary gold substrate. Li et al. employed a similar technique to successfully detect carcinoembryonic antigens in real human serum samples [73].

It is foreseen that both the recordable CD methodology and synthetic gold leaf methodology could find their way into a separation device, if a cost-effective and simplified synthetic protocol is established. The two, combined, have, however, inspired a recent concept within the field of centrifugal microfluidics. Centrifugal microfluidics induces fluid migration by actuating simple spinning mechanisms. However, electrodes must still be employed for separation of DNA fragments. Thompson et al. developed a gold leaf-based electrode using a fabrication technique derived from the PCL method (Figure 1g) [25]. Here, it was shown that commercially-available (beaten) gold leaf could be exploited, if stabilized on to polyester using a pressure sensitive adhesive. The resultant construct presents an option for electrode integration, which is remarkably inexpensive (100 s for less than $10) and easy to manufacture (Figure 1h). A notable advantage of these electrodes is that, unlike many of the preceding techniques, chemical reagents are not required. They can also be fabricated separately from the device itself, so are versatile in that they can be united with the primary device at a later date. This would, in turn, allow for an individual electrode design to be distributed to be compatible with microfluidic devices, much like Pt wire but at significantly lower cost. This model differs from that described earlier where electrodes are a part of the manufactured device itself. It is true, however, that if developers were to purchase electrode materials in this way, the onus would be on them to fabricate their devices so that both device and electrode were compatible. This would impact the choice of both substrate and optimal architecture. That being said, it is entirely feasible that both electrodes procured in this way and also those where the fabrication is incorporated, as part of the device manufacturing process could fit into the paradigm that dominates going forward. A summary of the methodologies discussed can be found in Table 2.

The majority of the examples discussed in this review are drawn from the academic domain, some of which may constitute part of a marketable product in later development. There are currently several leading commercial instruments available that offer a form of complete integrated genetic analysis. Cepheid^®^ (San Diego, CA, USA) offers a tool for clinical analysis which is supported by quantitative PCR and does not necessitate integrated electrodes [74]. Commercialized instruments for human identification are provided by LGC forensics (Teddington, UK); IntegeniX^®^, (Pleasanton, CA, USA); NEC (Tokyo, Japan); and NetBIO (Waltham, MA, USA). The LGC *ParaDNA* system is cored by a chemistry that involves fluorescent beacons that anneal with genomic targets and also do not require electrodes [75]. IntegeniX^®^, developer of the *RapidHIT*, implements DNA separation using a traditional fused silica capillary for separation, and an interfaced microfluidic device within the instrument for sample preparation (and PCR) [76]. By contrast, NEC, developer of the “*portable DNA analyzer*”, applies voltages to reservoirs attached to the device separation domain by the introduction of electrodes incorporated into the instrument lid [77]. Finally, NetBIO, developer of the *BioChipSet*™ has incorporated a conductive electrode strip (the composition remains proprietary), which can connect with a power supply via pogo pins at the edge of the device [78]. This electrode strip concept encapsulates many of the ideas presented throughout this review.

## 3. Conclusions

Looking ahead, we have learned (and continue to learn) from those who have integrated electrodes using photolithography, sputtering, evaporation, screen-printing, conductive polymers, etching, and gold leaf. It is likely that that much inspiration will be derived from the electrochemistry community, with additional innovations being offered by those developing supporting components such as resistive heaters or cell lysis actuators. To recapitulate, however, these features are not necessarily burdened with an additional preference for hermetic containment and/or disposability. Thus, the burden is largely on the developers to modify those methods accordingly.

The attractive characteristics showcased by the current commercial systems are speed, simplicity of use, and portability. The latter of these is defined by both size and weight. The expected deluge of instruments to support and compete with this pioneering generation are to be delivered by developers with the knowledge and expertise to tackle all of those essential attributes. In doing so, they can now dip into a melting pot rich with materials and functionalities which can likely be rearranged and assembled into novel, optimal systems that meet the need of the end-user. It is reassuring then, that the list of options for electrode integration is equally replete.

Finally, it has been emphasized previously [3] that developers must approach new fabrication challenges with a vision that acknowledges every aspect of their proposed device. While it has been demonstrated that integrating electrodes must be discussed at the inception of any microfluidic DNA separation project, this notion merely exemplifies that, now, no microfluidic endeavor should be pursued without adhering to an entirely holistic strategy.

## Figures and Tables

**Table 1:** Physical properties of popular electrode materials.

Material	Electrical Conductivity (10 × 10^6^ Siemens/m)	Electrical Resistivity (10 × 10^−8^ Ohm·m)	Density (g/cm^3^)	Melting Point or Degradation (°C)	Cost (USD/Lb)
Platinum	9.3	10.8	21.4	1772	14,915.52
Silver	62.1	1.6	10.5	961	269.28
Copper	58.5	1.7	8.9	1083	252
Gold	44.2	2.3	19.4	1064	19,526.4
Aluminum	36.9	2.7	2.7	660	0.79
Lithium	10.8	9.3	0.54	181	3.4
Carbon (ex polyacrylonitrile (PAN))	5.9	16.9	1.8	2500	10.5
Nickel	14.3	7	8.8	1455	5.31

**Table 2:** Summary of current methodologies for integrating electrodes in microfluidic devices.

Electrode Type	Ease of Fabrication	Cost of Fabrication (Per Device)	Issues	References
Wire	Easy	>$20	Difficult to integrate, often used with open device	[39,40,41,42]
Integrated wire	Difficult	>$20	Leakage issues	[43,44]
Sputter/evaporation	Difficult	>$10	Equipment requires trained personnel	[47,48,49,52,53]
Screen printed	Easy	<$1	Durability on some surfaces	[54,55]
Conductive polymer	Medium	<$5	Loss of conductivity at high voltages	[63,64,65]
Recordable CDs	Medium	<$1	Potential difficulties integrating with complex fluidics	[68,69,70]
Synthesized gold leaf	Difficult	<$1	Potential difficulties scaling synthetic protocol	[72,73]
Gold leaf	Easy	<10¢	Susceptible to damage upon contact	[25]

## References

[1] Whitesides G.M. (2006). The origins and the future of microfluidics. Nature.

[2] Ong S., Zhang S., Du H., Fu Y. (2008). Fundamental principles and applications of microfluidic systems. Front. Biosci..

[3] Chin C.D., Linder V., Sia S.K. (2012). Commercialization of microfluidic point-of-care diagnostic devices. Lab Chip.

[4] Price C.W., Leslie D.C., Landers J.P. (2009). Nucleic acid extraction techniques and application to the microchip. Lab Chip.

[5] Landers J.P. (2009). Molecular diagnostics on electrophoretic microchips. Anal. Chem..

[6] Bruijns B., van Asten A., Tiggelaar R., Gardeniers H. (2016). Microfluidic devices for forensic DNA analysis: A review. Biosensors.

[7] Sambrook J., Green M.R. (2012). Molecular Cloning: A Laboratory Manual.

[8] Butler J. (2005). Forensic DNA Typing.

[9] Wolfe K.A., Breadmore M.C., Ferrance J.P., Power M.E., Conroy J.F., Norris P.M., Landers J.P. (2002). Towards a microchip-based solid phase extraction method isolation of nucleic acid. Electrophoresis.

[10] Bishop D.H.L., Claybrook J.R., Spiegelman S. (1967). Electrophoretic separation of viral nucleic acids. J. Mol. Biol..

[11] Fangman W.L. (1978). Separation of very large DNA molecules by gel electrophoresis. Nucl. Acids Res..

[12] Carle G.F., Frank M., Olsen M.V. (1986). Electrophoretic separations of large DNA molecules by periodic inversion of the electric field. Science.

[13] Smithies O. (2012). How it all began: A personal history of gel electrophoresis. Methods Mol. Biol..

[14] Landers J.P. (1993). Capillary electrophoresis: Pioneering new approaches for biomolecular analysis. Trends Biochem. Sci..

[15] Grossman P.D., Colburn J.C. (1992). Capillary Electrophoresis: Theory and Practice.

[16] Manz A., Harrison D.J., Fettinger J.C., Verpoorte E., Ludi H., Widmer H.M. Integrated electroosmotic pumps and flow manifolds for total chemical analysis. Proceedings of the 1991 International Conference on Solid-State Sensors and Actuators (TRANSDUCERS ’91).

[17] Mcknight T.E., Culbertson C.T., Jacobson S.C., Ramsey J.M. (2001). Electroosmotically induced hydraulic pumping with integrated electrodes on microfluidic devices. Anal. Chem..

[18] Oakley J.A., Shaw K.J., Docker P.T., Dyer C.E., Greenman J., Greenway G.M., Haswell S.J. (2009). Development of a bi-functional silica monolith for electro-osmotic pumping and DNA clean-up/extraction using gel-supported reagents in a microfluidic device. Lab Chip.

[19] Martinez-Duarte R. (2012). Microfabrication technologies in dielectrophoresis applications—A review. Electrophoresis.

[20] Pethig R. (2010). Dielectrophoresis: Status of the theory, technology, and applications. Biomicrofluidics.

[21] Gui L., Ren C.L. (2006). Numeric simulation of heat transfer and electrokinetic flow in an electroosmotic-based continuous flow PCR chip. Anal. Chem..

[22] Laser D.J., Santiago J.G. (2004). A review of micropumps. J. Micromech. Microeng..

[23] Baldwin R.P., Roussel T.J., Crain M.M., Bathlagunda V., Jackson D.J., Gullapalli J., Conklin J.A., Pai R., Naber J.F., Walsh K.M. (2002). Fully Integrated On-Chip Electrochemical Detection for Capillary Electrophoresis in a Microfabricated Device. Anal. Chem..

[24] Lin B. (2011). Microfluidics: Technologies and Applications: Topics in Current Chemistry.

[25] Thompson B.L., Birch C., Nelson D.A., Li J., DuVall J.A., Le Roux D.S., Tsuei A., Mills D.L., Root B.E., Landers J.P. (2016). A centrifugal microfluidic device with integrated gold leaf electrodes for the electrophoretic separation of DNA. Lab Chip.

[26] Bakker E., Telting-Diaz M. (2002). Electrochemical Sensors. Anal. Chem..

[27] Rossier J., Reymond F., Michel P.E. (2002). Polymer microfluidic chips for electrochemical and biochemical analyses. Electrophoresis.

[28] Thiyagarajan N., Chang J., Senthilkumar K., Zen J. (2014). Disposable electrochemical sensors: A mini review. Electrochem. Commun..

[29] Cohen R.D. (2014). Movement and Motion.

[30] Kohlheyer D., Eijkel J.C.T., Schlautmann S., van den Berg A., Schasfoort R.B.M. (2008). Bubble-free operation of a microfluidic free-flow electrophoresis chip with integrated Pt electrodes. Anal. Chem..

[31] Tsai D.-M., Lin K.-W., Zen J.-M., Chen H.-Y., Hong R.-H. (2005). A new fabrication process for a microchip electrophoresis device integrated with a three-electrode electrochemical detector. Electrophoresis.

[32] Kohler S., Weilbeer C., Howitz S., Becker H., Beushausen V., Belder D. (2011). PDMS free-flow electrophoresis chips with integrated partitioning bars for bubble segregation. Lab Chip.

[33] (2010). ABI PRISM 310 Genetic Analyzer: User’s Manual.

[34] Kuschel M. (2000). Analysis of Messenger RNA Using the Agilent 2100 Bioanalyzer and the RNA 6000 LabChip^®^ Kit.

[35] (2009). Beckman Coulter^®^ Genome Lab™ Genetic Analysis System: User’s Guide.

[36] Zhuang B., Han J., Xiang G., Gan W., Wang S., Wang D., Wang L., Sun J., Li C., Liu P. (2016). fully integrated and automated microsystem for rapid pharmacogenetic typing of multiple warfarin related single-nucleotide polymorphisms. Lab Chip.

[37] Hopwood A.J., Hurth C., Yang J., Cai Z., Moran N., Lee-Edgehill J.G., Nordquist A., Lenigk R., Estes M.D., Haley J.P. (2010). Integrated microfluidic system for rapid forensic DNA analysis: Sample collection to DNA profile. Anal. Chem..

[38] Huang X.C., Quesada M.A., Mathies R.A. (1992). Capillary array electrophoresis using laser-excited confocal fluorescence detection. Anal. Chem..

[39] Harrison D.J., Manz A., Fan Z. (1992). Capillary electrophoresis and sample injection systems integrated on a planar glass chip. Anal. Chem..

[40] Harrison D., Fluri K., Seiler K., Fan Z., Effenhauser C.S., Manz A. (1993). Micromachining a miniaturized capillary electrophoresis-based chemical analysis system on a chip. Science.

[41] Woolley A.T., Mathies R.A. (1994). Ultra-high-speed DNA fragment separations using microfabricated capillary array electrophoresis chips. Proc. Nati. Acad. Sci. USA.

[42] Wooley A.T., Hadley D., Landre P., deMello A.J., Mathies R.A., Northrup M.A. (1996). Functional integration of PCR amplification and capillary electrophoresis in a microfabricated DNA analysis device. Anal. Chem..

[43] McCormick R.M., Nelson R.J., Alonso-Amigo M.G., Benvegnu D.J., Hooper H.H. (1997). Microchannel Electrophoretic Separations of DNA in Injection-Molded Plastic Substrates. Anal. Chem..

[44] Sanders J., Breadmore M.C., Mitchell P.S., Landers J.P. (2002). A simple PDMS-based electro-fluidic interface for microchip electrophoretic separations. Analyst.

[45] Zhong M., Lunte S.M. (1996). Integrated On-Capillary Electrochemical Detector for Capillary Electrophoresis. Anal. Chem..

[46] Voegel P.D., Zhou W., Baldwin R.P. (1997). Integrated capillary electrophoresis electrochemical detection with metal film electrodes directly deposited onto the capillary tip. Anal. Chem..

[47] Burns M.A., Johnson B.N., Brahmasandra S.N., Handique K., Webster J.R., Krishnan M., Sammarco T.S., Man P.M., Jones D., Heldsinger D. (1998). An integrated Nanoliter DNA Analysis Device. Science.

[48] Woolley A.T., Lao K., Glazer A.N., Mathies R.A. (1998). Capillary Electrophoresis Chips with Integrated Electrochemical Detection. Anal. Chem..

[49] Lagally E.T., Scherer J.R., Blazej R.G., Toriello N.M., Diep B.A., Ramchandani M., Sensabaugh G.F., Riley L.W., Mathies R.A. (2004). Integrated Portable Genetic Analysis Microsystem for Pathogen/Infectious Disease Detection. Anal. Chem..

[50] Floris A., Staal S., Lenk S., Staijen E., Kohlheyer D., Eijkel J., van den Berg A. (2010). A prefilled, ready-to-use electrophoresis based lab-on-a-chip device for monitoring lithium in blood. Lab Chip.

[51] Ren K., Zhou J., Wu H. (2013). Materials for microfluidic fabrication. Acc. Chem. Res..

[52] Jha S.K., Chand R., Han D., Jang Y., Ra G., Kim J.S., Nahmb B., Kim Y. (2012). An integrated PCR microfluidic chip incorporating aseptic electrochemical cell lysis and capillary electrophoresis amperometric DNA detection for rapid and quantitative genetic analysis. Lab Chip.

[53] Le Roux D., Root B.E., Hickey J.A., Scott O.N., Tsuei A., Li J., Saul D.J., Chassagne L., Landers J.P., de Mazencourt P. (2014). An integrated sample-in-answer-out microfluidic chip for rapid human identification by STR analysis. Lab Chip.

[54] Koh C.G., Tan W., Zhao M., Ricco A.J., Hugh Fan Z. (2003). Integrating polymerase chain reaction, valving and electrophoresis in a plastic device for bacterial detection. Anal. Chem..

[55] Liu Y., Li C., Li Z., Chan S.D., Eto D., Wu W., Zhang J.P., Chien R.-L., Wada H.G., Greenstein M. (2016). On-chip quantitation PCR using integrated real-time detection by capillary electrophoresis. Electrophoresis.

[56] Shaw K.J., Joyce D.A., Docker P.T., Dyer C.E., Greenway G.M., Greenman J., Haswell S.J. (2011). Development of a real-world direct interface for integrated DNA extraction and amplification in a microfluidic device. Lab Chip.

[57] Krebs F.C. (2009). Fabrication and processing of polymer solar cells: A review of printing and coating techniques. Sol. Energy Mater. Sol. Cells.

[58] Taleat Z., Khoshroo A., Mazloum-Ardakani M. (2014). Screen-printed electrodes for biosensing: A review (2008–2013). Microchim. Acta.

[59] Nie Z., Nijhuis C.A., Gong J., Chen X., Kumachev A., Martinez A.W., Narovlyansky M., Whitesides G.M. (2010). Electrochemical sensing in paper-based microfluidic devices. Lab Chip.

[60] Li X., Ballerini D.R., Shen W. (2012). A perspective on paper-based microfluidics: Current status and future trends. Biomicrofluidics.

[61] Dungchai W., Chailapukal O., Henry C.S. (2009). Electrochemical Detection for Paper-Based Microfluidics. Anal. Chem..

[62] Manz A., Effenhauser C.S., Burggraf N., Harrison D.J., Seiler K., Fluri K. (1993). Electroosmotic pumping and electrophoretic separations for miniaturized chemical analysis systems. J. Micromech. Microeng..

[63] Bengtsson K., Nilsson S., Robinson N.D. (2014). Conducting polymer electrodes for gel electrophoresis. PLoS ONE.

[64] Henderson R.D., Guijt R.M., Haddad P.R., Hilder E.F., Lewis T.W., Breadmore M.C. (2010). Manufacturing and application of a fully polymeric electrophoresis chip with integrated polyaniline electrodes. Lab Chip.

[65] Li D., Huang J., Kaner R.B. (2009). Polyaniline Nanofibers: A Unique Polymer Nanostructure for Versatile Applications. Acc. Chem. Res..

[66] Do Lago C.L., da Silva H.D., Neves C.A., Brito-Neto J.G., da Silva J.A. (2003). A dry process for production of microfluidic devices based on the lamination of laser-printed polyester films. Anal. Chem..

[67] Thompson B.L., Ouyang Y., Duarte G.R.M., Carrilho E., Krauss S.T., Landers J.P. (2015). Inexpensive, rapid protoyping of microfluidic devices using overhead transparencies and laser print, cut and laminate fabrication method. Nat. Protoc..

[68] Angnes L., Richter E.M., Augelli M.A., Gustavo G.H. (2000). Gold electrodes from recordable CDs. Anal. Chem..

[69] Eduardo M Richter E.M., Augelli M.A., Kume G.H., Mioshi R.N., Angnes L. (2000). Gold electrodes from recordable CDs for mercury quantification by flow injection analysis. Anal. Bioanal. Chem..

[70] Daniel D., Gutz I.G.R. (2003). Quick production of gold electrode sets or arrays and of microfluidic flow cells based on heat transfer of laser printed toner masks onto compact disks. Electrochem. Commun..

[71] Foguel M.V., dos Santos G.P., Ferreira A.A.P., Magnani M., Mascini M., Skladal P., Benedetti A.V., Yamanaka H. (2016). Comparison of Gold CD-R Types as Electrochemical Device and as Platform for Biosensors. J. Braz. Chem. Soc..

[72] Ciesielski P.N., Scott A.M., Faulkner C.J., Berron B.J., Cliffel D.E., Jennings G.K. (2008). Functionalized nanoporous gold leaf electrode films for the immobilization of photosystem I. ACS Nano.

[73] Li X., Wang R., Zhang X. (2011). Electrochemiluminescence immunoassay at a nanoporous gold leaf electrode and using CdTe quantun dots as labels. Microchim. Acta.

[74] (2012). Genexpert Dx System: Operator Guide.

[75] Blackman S., Dawnay N., Ball G., Stafford-Allen B., Tribble N., Rendall P., Neary K., Hanson E.K., Ballantyne J., Kallifatidis B. (2015). Developmental validation of the ParaDNA1 intelligence system—A novel approach to DNA profiling. Forensic. Sci. Int. Genet..

[76] Jovanovich S., Bogdan G., Belcinski R., Buscaino J., Burgi D., Butts E.L.R., Chear K., Ciopyk B., Eberhart D., El-Sissi O. (2015). Developmental validation of a fully integrated sample-to-profile rapid human identification system for processing single-source reference buccal samples. Forensic. Sci. Int. Genet..

[77] NEC: Portable DNA. http://th.nec.com/en_TH/solution/safetysocial/portabledna.htmlx.

[78] Turingan R.S., Vasantgadkar S., Palombo L., Hogan C., Jiang H., Tan E., Selden R.F. (2016). Rapid DNA analysis for automated processing and interpretation of low DNA content samples. Investig. Genet..

